# Hsa_circ_0000081 promotes the function of gastric cancer through sponging hsa-miR-423-5p to influence 3-phosphoinositide-dependent kinase 1 expression

**DOI:** 10.1080/21655979.2022.2053796

**Published:** 2022-03-18

**Authors:** Fei Jiang, Xueju Hu, Hongyong Cao, Xiaobing Shen

**Affiliations:** a Key Laboratory of Environmental Medical Engineering and Education Ministry, Nanjing Public Health College, Southeast University, Nanjing, China; b Department of Epidemiology and Health Statistics, School of Public Health, Southeast University, Nanjing, China; c Department of Occupational and Environmental Health, School of Public Health, Southeast University, Nanjing, China; d Department of General Surgery, Nanjing First Hospital, Nanjing Medical University, Nanjing, China

**Keywords:** circRNA, miRNA, gastric cancer, mRNA, ceRNA

## Abstract

Gastric cancer (GC) is one of the most common malignancies in the world, and effective therapeutic targets need to be identified for this type of cancer. In this study, circular RNA (circRNA) microarray analysis was utilized to screen differentially expressed circRNA in GC. Using quantitative reverse transcription polymerase chain reaction (qRT-PCR), hsa_circ_0000081 (circRNA-0000081) expression was found to be up-regulated in tissues and cells and was negative correlated with patients’ survival time. RNase R and Actinomycin D assays indicated that circRNA-0000081 was significantly more resistant to R enzyme and had a longer half-life than linear RNA. Moreover, the knockdown or overexpression of circRNA-000081 could influence the proliferation, migration, and invasion potential of GC. Finally, dual luciferase reporter, RNA immunoprecipitation, qRT-PCR, and western blotting assays were used to verify the targeting relationship between circRNA-000081 and miRNA-423-5p or miRNA-423-5p and 3-phosphoinositide-dependent kinase 1 (PDPK1). In conclusion, circRNA-0000081 promotes the function of GC through sponging hsa-miR-423-5p to influence PDPK1 expression, which has a promising therapeutic potential for treating patients with GC.

## 1 Introduction

Gastric cancer (GC) remains one of the most common types of malignant tumors that burden healthcare systems worldwide. Over one million new cases were reported and an estimated 769,000 GC-related deaths occurred in 2020, ranking fifth in incidence and fourth in mortality globally [1]. Most patients with GC are diagnosed at an advanced stage [2]; the age-standardized five-year survival for gastric cancer was reported to be below 30% in most countries [3]. Currently, common treatment methods are surgery, radiotherapy, chemotherapy, or a combination of these [4]. Although these methods improve the survival rate of patients, radiotherapy is limited by its lack of targeting ability, chemotherapy has toxic and side effects, and surgical treatment is invasive [5]. Therefore, there is an urgent need to identify early diagnostic markers and effective treatment targets.

In recent years, circular RNA (circRNA), which differs from linear RNA with its covalently closed structure, has attracted a lot of attention for its role in cancer diagnosis and treatment [6]. For example, circ3823 was more stable than PVT1, promoting colorectal cancer growth, metastasis, and angiogenesis through the circ3823/miR-30c-5p/TCF7 axis, and it may serve as a new diagnostic marker or therapeutic target for the treatment of colorectal cancer patients [7]. In addition, circRNAs can be very highly expressed and conserved among species. Moreover, these molecules often showed tissue-specific or developmental-stage-specific expression [8]. For instance, hsa_circ_0044235 and hsa_circ_0068367 expression was found to be significantly down-regulated in patients with systemic lupus erythematosus and hence can play a significant role in systemic lupus erythematosus diagnosis [9]. Similarly, circSHKBP1 was found to be highly abundant in many endothelial cells [10], and it was characterized as a promising circulating biomarker for GC diagnosis and prognosis and an exceptional candidate for further therapeutic exploration [11].

MicroRNAs (miRNAs) are a class of short-length non-coding single-stranded RNA (up to 23 nucleotides). They act as post-transcriptional regulators of genes [12], which is also a potential research direction for cancer treatment and diagnosis. For example, miR-21 could serve as a therapeutic target or early stage prognostic biomarker for lung adenocarcinoma, as it is associated with disease progression and survival [13]. In a similar fashion, miR-30b-5p and miR-99a-5p could serve as diagnostic biomarkers for breast cancer [14]. X Zhang et al. found that hsa-miR-375 and hsa-miR-142-5p were involved in regulating target genes in several oncogenic signal pathways and could serve as predictors of disease progression in GC [15].

Furthermore, the main mechanism through which circRNA participated in the development of cancers is by acting as a molecular sponge for miRNA. For example, circDIDO1ʹs regulation of hepatic stellate cells requires the participation of mir-141-3p [16]. Similarly, circRNA_0067934 promotes glioma development with the help of microRNA miR-7 [17], and hsa_circ_0008896 accelerates atherosclerosis in a process that requires the participation of hsa-miR-633 [18].

In this study, we aimed to elucidate the association between circRNA-0000081 and GC patients’ survival time and to clarify the regulatory mechanism of the circRNA-0000081/miRNA-423-5p/PDPK1 axis in GC. We hypothesize that circRNA-0000081 participates in the development of GC through the circRNA-0000081/miRNA-423-5p/PDPK1 axis. To the best of our knowledge, this is the first study on circRNA-0000081. And the purpose of this study is to provide a basis for the clinical transformation of molecular therapy for GC.

## 2 Materials and methods

### 2.1 Tissues

We collected 61 pairs of GC tissues and adjacent normal tissues from patients in the Nanjing No. 1 City Hospital from 2015 to 2016. All specimens were adequately stored at −80°C until RNA extraction. Informed consent was obtained from each patient before enrolling them in the study. The study design was approved by the Ethics Committee of Nanjing Medical University.

### 2.2 Cells

MKN-28 (MKN-74), MKN-45, AGS, MGC-803, and GES-1 cell lines were used and with their own STR (short tandem report) information report. The RPMI (Roswell Park Memorial Institute) 1640 culture medium, containing 10% fetal bovine serum and 1% penicillin and streptomycin (Gibco Life Technologies, USA), was used for all cell lines. All cells were cultured with 5% CO_2_ at 37°C [19].

### 2.3 Quantitative reverse transcription polymerase chain reaction (qRT-PCR)

TRIzol reagent was used to extract total RNA from tissues and cells (GenStar, China). The cDNA of circRNA, miRNA, and mRNA was synthesized using a reverse transcription kit (GenStar, China, and Takara Bio, Japan). The quantification of circRNA, miRNA, and mRNA was performed using a SYBR Green PCR Kit (Yeasen Biotech Co., Ltd, China). CircRNA sequences were designed and synthesized by GenePharma (Shanghai, China) with a specific melting curve, smooth amplification curve, and electrophoresis diagram corresponding to the fragment length. The miRNA and mRNA sequences were synthesized by Generay Biotech Co. Ltd. (Nanjing, China). Glyceraldehyde 3-phospate dehydrogenase was used to normalize mRNA and circRNA expression, and U6 was used to normalize miRNA expression [20]. The primers could be found in Table S1.

### 2.4 RNase R assay

We measured the concentration of RNA, after extracting the total cell RNA, treating the total RNA with a specific concentration of RNase R, and finally, using qRT-PCR to explore the stability variations of circRNA-0000081 and linear RNA [21].

### 2.5 Actinomycin D

We seeded the AGS cells into a 24-well plate, with 15 × 10^4^ cells in each well. The cells were then treated with 1 µM actinomycin D1 µM after 24 hours. Next, the half-life variations of circRNA-0000081 and linear RNA were explored using qRT-PCR after 0 h, 4 h, 8 h, 12 h, and 24 h [22].

### 2.6 RNA fluorescence in situ hybridization (FISH)

An oligonucleotide-modified probe sequence of circRNA-0000081 was synthesized by Ribo Bio Technology Co Ltd (Guangzhou, China). In brief, we used the Dy3-marked circRNA-0000081 probe fixed by 4% paraformaldehyde. The fixed cells were permeabilized with 0.5% Triton X-100. After hybridizing, we utilized fluorescence confocal microscopy (Zeiss, Germany) to capture the images (DAPI was used to dye the nucleus of the cell) [22].

### 2.7 Cell counting kit-8 (CCK-8) proliferation assay

GC cells were cultured in 96-well plates at a density of 5000 cells per unit volume and treated with 10 μL CCK-8 solution per well after 22.5 h, 46.5 h, and 70.5 h (Meilun Biotechnology Co., LTD., Dalian, China). The absorbance of the cells was measured using microplates at a wavelength of 450 nm according to manufacturer’s instructions (Synergy4; BioTek, Winooski, VT, USA) [23].

### 2.8 Transwell assays

The Transwell assays were performed on a 24-well plate using Matrigel matrix glue (Corning, USA) (diameter 8 μm; Jet Bio-Filter Co., Guangzhou, China). GC cells were seeded in the upper chambers using siRNA or nc-siRNA (RIBO Bio, Guangzhou, China), pLV-circRNA-0000081 or pNC-circRNA-0000081 (HAN Bio, Shanghai, China), mimics-miRNA-423-5p or nc-mimics (HAN Bio, Shanghai, China), and inhibitor-miRNA-423-5p or nc-inhibitor (HAN Bio, Shanghai, China) in 200 μl of serum-free medium. The lower chambers were 600ul serum-containing medium. After incubation for 24 h, the cells passing through the well were fixed and stained with crystal violet (Solarbio, China) for 15 minutes. Finally, the cells were photographed and counted in three different areas [24].

### 2.9 Microarray analysis and ceRNA network analysis

Three pairs of GC tissues and adjacent normal tissues were evaluated to investigate potential key circRNAs in GC using the circRNA microarray by Kang Chen Biotech (China). Briefly, circular RNAs were amplified and transcribed into fluorescent cRNA utilizing a random priming method (Arraystar Super RNA Labeling Kit; Arraystar). The labeled cRNAs were hybridized onto Arraystar Human circRNA Array V2 (8x15K, Arraystar). After the slides were washed, the arrays were scanned with an Agilent Scanner G2505C. The target miRNAs were predicted based on Cancer-Specific CircRNA Database, while targeted mRNAs were predicted based on TargetScan, miRDB, and miRTarBase [17].

### 2.10 RNA immunoprecipitation (RIP) assay

According to the manufacturer’s instructions, firstly, AGS cells (approximately 1 × 10^7^) were washed with cold PBS twice and lysed with RIP lysis buffer (EMD Millipore, Billerica, MA, U.S.A). Then, the cell lysates were incubated with RIP immunoprecipitation buffer containing magnetic beads conjugated with human anti-Argonaute2 (AGO2) or PDPK1 antibody (Rabbit antibodies, ABclonal, China) or negative control rabbit IgG (Millipore, Billerica, MA, U.S.). Samples were incubated with Proteinase K, and immunoprecipitated RNA was isolated. Extracted RNAs were analyzed by qRT-PCR to determine whether circRNA-0000081 and miRNA-423-5p were pulled down by the target protein [25].

### 2.11 Dual luciferase reporter assay

Wild-type (WT) and mutant (MUT) fragments of the 3`-UTR of circRNA-0000081 related to the miRNA-423-5p binding site were designed, synthesized, and inserted into the pSI-Check2 vector (Hanheng, Shanghai, China). The pSI-Check2 vectors carrying circRNA-0000081-WT or circRNA-0000081-MUT and miRNA-423-5p mimics or negative control (nc) were cotransfected into 293 T-cells. After 48 h, luciferase activity in the cotransfected cells was detected using the dual luciferase reporter assay system according to the manufacturer’s instructions [26].

### 2.12 Western blotting (WB)

Cells were lysed in RIPA lysis buffer (RIPA, Beyotime, China). The proteins were quantified by bicinchoninic acid analysis (GenStar, China). Equal amounts of protein were separated by 7.5% or 12.5% SDS-PAGE (EpiZyme Bio, Shanghai, China) and transferred onto a PVDF (polyvinylidene fluorid) membrane (Millipore, USA). The membrane was blocked with 5% skim milk powder and incubated with primary antibodies against PDPK1 (Rabbit antibodies, ABclonal, China) and actin (Cell Signaling Technology, USA) at 4°C for more than 8 h. The membranes were then incubated with the secondary antibody (1:5000) for 1 h. Finally, the blots were visualized by Chemiluminescent HRP Substrate (Millipore, U.S.) and analyzed by ImageJ (National Institutes of Health, USA) [27].

### 2.13 Statistical analysis

Prism 6 and R software were used to analyze all data. The paired samples t-test or two independent samples t-test was used to analyze the data between two groups. Kaplan-Meier and log-rank tests were used to compare the relationship between circRNA-0000081 expression and patients’ survival. The data were analyzed and visualized using R software’s survival and survminer packages. *P* values less than 0.05 were considered statistically significant, except in the survival statistical analysis of circRNA-0000081; *P* values less than 0.1 were considered statistically significant due to the small sample size.

## 3 Results

### 3.1 Relationship betweencircRNA-0000081 expression and patient survival

**Figure 1.**
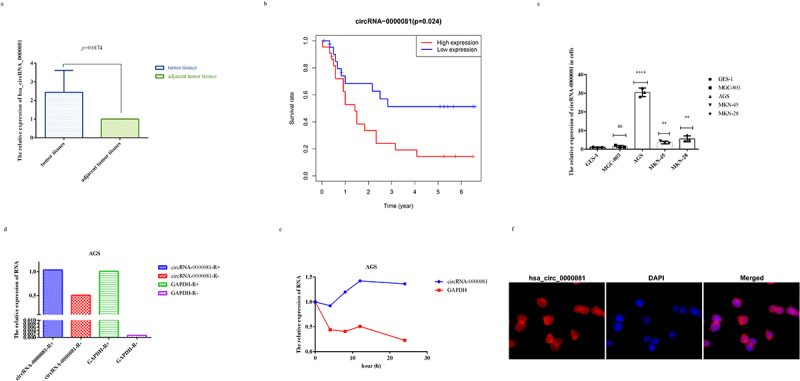
A. The relative expression of circRNA-0000081 in 61 pair gastric cancer tissues (paired sample t-test was performed using the 2^−ΔΔCT^ value of each pair of samples); b. Survival analysis of patients with different expression levels of circRNA-0000081; c. QRT-PCR of circRNA-0000081 in 6 gastric cell lines; d. R RNase assay results of circRNA-0000081 in AGS; e. Actinomycin D assay results of circRNA-0000081 in AGS; f. FISH assay results of circRNA-0000081 in AGS (The samples were imaged at 400* magnification).

We performed microarray sequencing on three pairs of GC tissue samples, and 11,514 circRNAs were identified, 5,775 of which were up-regulated and 5,739 down-regulated. There were 25 circRNAs with significant differences in expression (circRNAs with absolute fold ratio greater than 2 and *P* value less than 0.05 were called circRNAs with significant differences in expression), among which 13 were up-regulated and 12 were down-regulated (Fig S1a). After preliminary tissue analysis, the downregulation of circRNA-0000081 expression was consistent with the sequencing results (Fig S1b, n = 30); however, further analysis of the larger sample showed that the expression of circRNA-0000081 was up-regulated (Figure 1a, n = 61). In fact, the higher the expression of circRNA-0000081, the shorter the survival time of the patients (Figure 1b, n = 45), while there was no significant correlation between circRNA-0000081 expression and age, gender, stage, tumor size, and other factors (Table 1).

**Table 1.** Analysis of the correlation between circRNA-0000081 expression and clinicopathological parameters

			CircRNA-0000081 expression		
Characteristics	Variable	Number(61)	low	high	*p (X^2^ test)*
Age(years)	≤65	21	12	9	0.3614
	>65	38	17	21	
	NA	2			
Gender	female	21	10	11	0.861
	male	38	19	19	
	NA	2			
AJCC(Version Type 7th)	stage I+ II stage III+IV	5 + 23 27 + 3	2 + 14 11 + 2	3 + 9 16 + 1	0.2932
	NA	3			
Tumor size (cm)	<=4.5	30	14	16	0.6958
	>4.5	27	14	13	
	NA	4			
Family history	yes	3	2	1	0.5255
	no	44	21	23	
	NA	14			
History of hypertension	yes	26	13	13	1
	no	28	14	14	
	NA	7			
Blood type	A	18	11	7	0.3711
	AB	5	1	4	
	B	10	4	6	
	O	21	11	10	
	NA	7

After that, we investigated the expression levels of circRNA-0000081 in GC cell lines and gastric epithelial cells. The results indicated that the expression of circRNA-0000081 was significantly up-regulated in GC cells than in gastric epithelial cells, which is consistent with the verification results that we obtained after sample enlargement (Figure 1c).

To explore whether circRNA-0000081 has higher stability than linear RNA, we treated AGS cells with R RNase and Actinomycin D. Results showed that circRNA-0000081 was significantly more resistant to R enzyme and had a longer half-life than linear RNA, which is consistent circRNA characteristics (Figure 1(d,e)). Moreover, the results of the FISH assay indicated that circRNA-0000081 mainly existed in the cytoplasm (Figure 1f). These results show that circRNA-0000081 is correlated with the survival time of GC patients, is more stable than linear RNA, and mainly exists in the cytoplasm, which all make it worthy of further investigation.

### 3.2 The function of circRNA-0000081 in vitro

**Figure 2.**
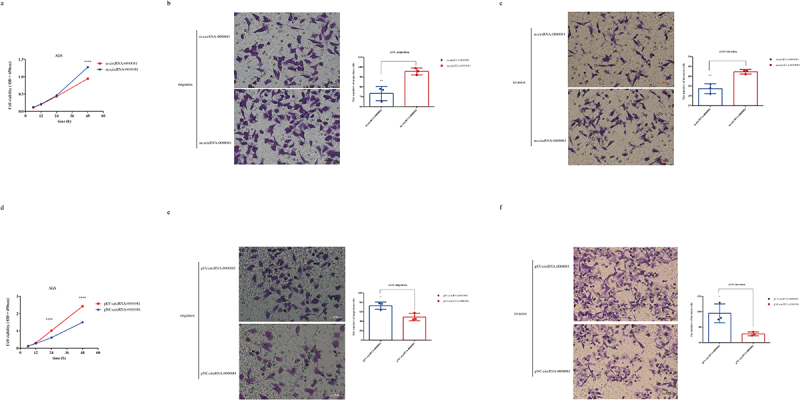
A. CCK-8 assay of AGS cells in the si-circRNA-0000081 group and nc-circRNA-0000081 group; b. Migration results of the Transwell assay of the si-circRNA-0000081 group and nc-circRNA-0000081 group (The samples were imaged at 200* magnification. Scale bar = 50 μm); c. Invasion results of the Transwell assay of AGS cells transfected with si-circRNA-0000081 and nc-circRNA-0000081 (The samples were imaged at 200* magnification. Scale bar = 50 μm); d. CCK-8 assay of AGS cells in the pLV-circRNA-0000081 group and pNC-circRNA-0000081 group; e. Migration results of the Transwell assay of the pLV-circRNA-0000081 group and pNC-circRNA-0000081 group (The samples were imaged at 200* magnification. Scale bar = 50 μm); f. Invasion results of the Transwell assay of AGS cells transfected with pLV-circRNA-0000081 and pNC-circRNA-0000081 (The samples were imaged at 200* magnification. Scale bar = 50 μm).

As FISH analysis showed that circRNA-0000081 mainly existed in the cytoplasm (Figure 1f), we constructed a cell line with silenced circRNA-0000081 using small interfering RNA (siRNA) (Fig S2a). CCK8 and Transwell experiments were then performed in cell lines successfully constructed with circRNA-0000081 knockdown. Results showed that knocking down circRNA-0000081 could significantly inhibit the proliferation, migration, and invasion of GC cells (Figure 2a, 2b and 2c).

Furthermore, another cell line with overexpressed circRNA-0000081 was successfully constructed (Fig S2b). CCK8 and Transwell assays were conducted and showed that the overexpression of circRNA-0000081 significantly increased the proliferation, migration, and invasion of GC cells (Figure 2d, 2e and 2 f).

These results indicated that circRNA-0000081 had a significant cancer-promoting effect. So, we speculate that circRNA-0000081 maybe involved in the development of GC through specific underlying molecular mechanisms that are yet to be elucidated.

### 3.3 CircRNA-0000081 could act as a molecular sponge of miRNA-423-5p

**Figure 3.**
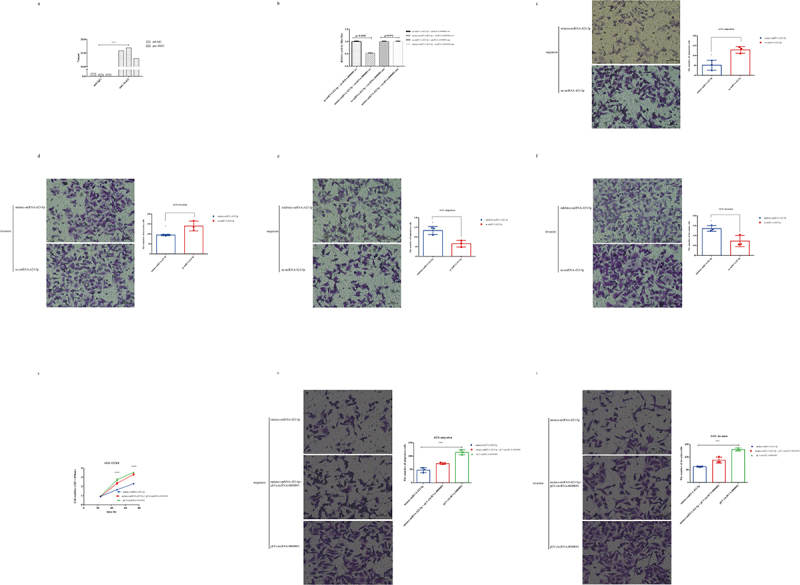
A. AGO2-RIP assay results of circRNA-0000081 in AGS; b. Luciferase reporter results for miRNA-423-5p and circRNA-0000081; c. Migration results of the Transwell assay of the mimics-miRNA-423-5p group and nc-miRNA-423-5p group (The samples were imaged at 200* magnification. Scale bar = 50 μm); d. Invasion results of the Transwell assay of AGS cells transfected with mimics-miRNA-423-5p group and nc-miRNA-423-5p group (The samples were imaged at 200* magnification. Scale bar = 50 μm); e. Migration results of the Transwell assay of the inhibitor-miRNA-423-5p group and nc-miRNA-423-5p group (The samples were imaged at 200* magnification. Scale bar = 50 μm); f. Invasion results of the Transwell assay of AGS cells transfected with inhibitor-miRNA-423-5p group and nc-miRNA-423-5p group (The samples were imaged at 200* magnification. Scale bar = 50 μm); g. CCK8 rescue results of mimics-423-5p group, mimics-423-5p+pLV-circRNA-0000081 group and pLV-circRNA-0000081 group; h. Migration rescue results of the Transwell assay of mimics-423-5p group, mimics-423-5p+pLV-circRNA-0000081 group, and pLV-circRNA-0000081 group (The samples were imaged at 200* magnification. Scale bar = 50 μm); i. Invasion rescue results of the Transwell assay of mimics-423-5p group, mimics-423-5p+pLV-circRNA-0000081 group, and pLV-circRNA-0000081 group (The samples were imaged at 200* magnification. Scale bar = 50 μm).

Since the current reports on the mechanism of mainly existing in the cytoplasm circRNAs is as a sponge for miRNAs, we explored whether circRNA-0000081 could be pulled down by AGO2 antibody through AGO2-RIP experiment. Results showed that AGO2 antibody could significantly pull down circRNA-0000081 when compared with that by the anti-IgG group (Figure 3a, *p*= 0.0002).

Using the software miRnada, we predicted that circRNA-0000081 could target more than 50 miRNAs (Table S1), among which miRNA-423-5p was reported to be associated with a variety of cancers. So, we selected miRNA-423-5p for further analysis.

The dual luciferase reporter assay showed a direct correlation between miRNA-423-5p and circRNA-0000081. We further found that miRNA-423-5p mimics (mimics-miRNA-423-5p) could inhibit the activity of circRNA-0000081 wt (wt-circRNA-0000081), while it could not influence the activity of circRNA-0000081 mu (mu-circRNA-0000081) (Figure 3b). At the RNA level, there was a significant negative correlation between their expression (Fig S3a).

Considering that there are few studies on miRNA-423-5p in GC, we used specific mimickers (Fig S3b) and inhibitors (Fig S3c) to construct cell lines with miRNA-423-5p silencing and overexpression, respectively, to explore the role of miRNA-423-5p in GC cells. Transwell assays showed that the overexpression of miRNA-423-5p could inhibit the migration and invasion of GC cells (Figure 3c and 3d). Whereas, the inhibition of miRNA-423-5p expression promoted the migration and invasion of GC cells (Figure 3e and 3 f).

When we treated cells with overexpressed circRNA-0000081 (plv-circRNA-0000081) and overexpressed miRNA-423-5p (mimics-miRNA-423-5p), it was found that plv-circRNA-0000081 could partially restore the inhibitory effects of mimics-miRNA-423-5p on cell proliferation (Figure 3g), migration (Figure 3h), and invasion (Figure 3i). In addition, mimics-miRNA-423-5p could partially restore the promoting effects of plv-circRNA-0000081 on cell proliferation (Figure 3g), migration (Figure 3h), and invasion (Figure 3i).

The above experimental results indicated that miRNA-423-5p had a certain anti-cancer effect on GC cells, and circRNA-0000081 could act as a molecular sponge of miRNA-423-5p and influenced the effect of miRNA-423-5p on GC cells.

### 3.4 The association between PDPK1 and circRNA-0000081

**Figure 4.**
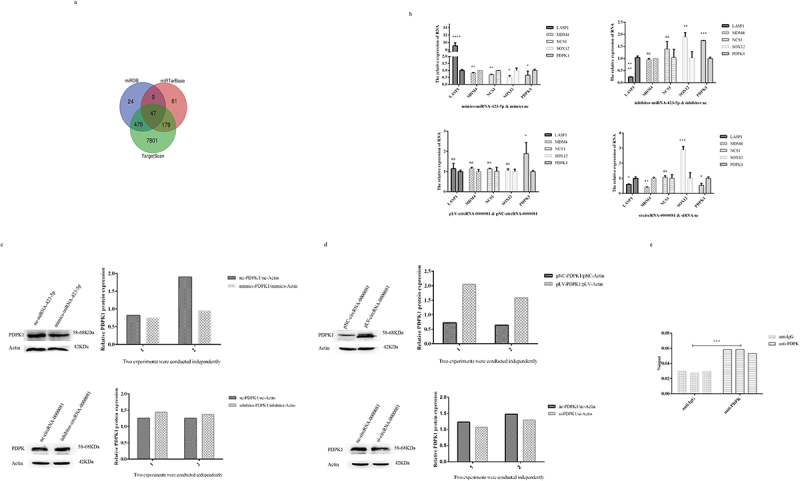
A. The targeted genes of miRNA-423-5p; b. QRT-PCR results of LASP1, MDM4, NCS1, SOX12, and PDPK1 in cells transfected with mimics-miRNA-423-5p, inhibitor-miRNA-423-5p, pLV-circRNA-0000081, si-circRNA-0000081, and their control groups; c. WB results of PDPK1 in cells transfected with mimics-miRNA-423-5p, inhibitor-miRNA-423-5p, pLV-circRNA-0000081, si-circRNA-0000081, and their control groups; d. Rescue experiment of PDPK1 protein; e. RIP experimental results of miRNA-423-5p pulled down by PDPK1 antibody.

After confirming that circRNA-0000081 could act as a molecular sponge of miRNA-423-5p, we predicted the downstream target of miRNA-423-5p using miRDB, miTarbase, and TargetScan software. Forty-seven intersecting mRNAs were found (Figure 4a).

Basing on the available literature, five mRNAs, LASP1, MDM4, NCS1, SOX12, and PDPK1, were selected for preliminary investigation at the RNA level. It was found that only PDPK1 was negatively correlated with miRNA-423-5p and positively correlated with circRNA-0000081 (Figure 4b). Therefore, we chose PDPK1 for further investigation of this association at the protein level. Results showed that the expression of PDPK1 was significantly negatively correlated with levels of miRNA-423-5p and positively correlated with levels of circRNA-0000081, similar to the results at the RNA level (Figure 4c). Moreover, pLV-circRNA-0000081 could partially restore the inhibition of PDPK1 by mimics-miRNA-423-5p, and the promotion of PDPK1 by pLV-circRNA-0000081 could also be partially restored by mimics-miRNA-423-5p (Figure 4d). Following that, through RIP experiment, we found that PDPK1 antibody could pull down miRNA-423-5p, indicating that there was indeed an interaction between miRNA-423-5p and PDPK1 (Figure 4e). These results indicated that circRNA-0000081 participated in the development of GC by regulating PDPK1. This regulation is mediated by circRNA-0000081ʹs action as a sponge for miR-423-5p.

### 3.5 Effect of up-regulation of circrNA-0000081 on GC cell progression

**Figure 5.**
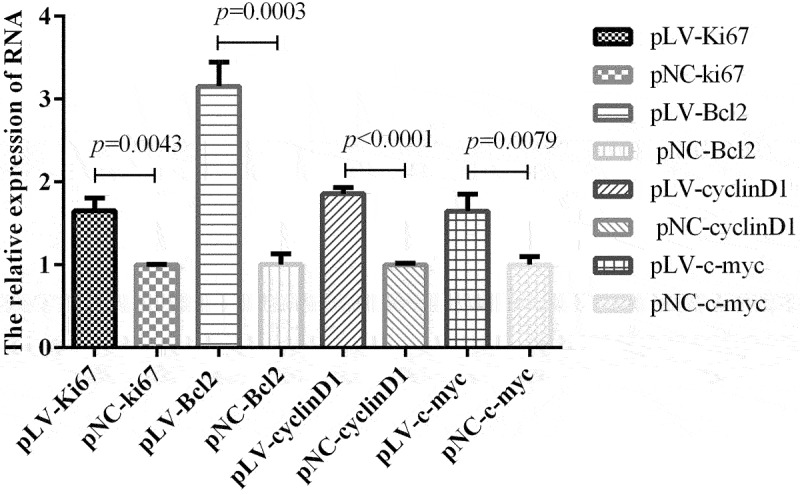
In the circrNA-0000081 overexpression model, the expression of key genes involved in proliferation, apoptosis, cell cycle, and oncogenesis.

In view of the role of circrNA-0000081 in cells and the above mechanisms, we were curious about whether the expression of key genes related to cell proliferation, cell apoptosis, and cell cycle, as well as oncogenes, could be affected by the overexpression of circRNA-0000081. Therefore, we selected representative genes involved in proliferation, apoptosis, cell cycle, and cancer-promoting effects to explore this influence. On the premise of up-regulation of circrNA-0000081, mRNA expression of Ki67 (key gene of cell proliferation, pLV-Ki67 and pNC-Ki67), Bcl2 (key gene of cell apoptosis, pLV-Bcl2 and pNC-Bcl2), cyclin D1 (key gene of cell cycle, pLV-cyclin D1 and pNC-cyclin D1), and c-myc (oncogene, pLV-c-myc and pNC-c-myc) were detected, as shown in Figure 5a. When circrNA-0000081 expression was up-regulated, all four genes were significantly up-regulated. This result suggested that circRNA-0000081 may additionally affect the progression of GC by influencing these key genes of proliferation, apoptosis, and cell cycle, which provides us with a new research direction for the mechanism of circRNA’s role in cancer and further demonstrates the potential of circrNA-0000081 as a therapeutic target in GC.

## 4 Discussion

According to the Global Cancer Statistics 2018 and 2020, GC ranks fifth in mortality, and third and fourth in incidence, respectively [1,28], thus imposing an enormous burden on patients. Therefore, it is particularly important to discover early specific and sensitive indicators and effective therapeutic targets for clinical application [29].

CircRNA has become an attractive research domain as a cancer biomarker or therapeutic target due to its high stability [30], abundance [31], and specificity of expression [32]. For instance, circPTPN22 might be a novel biomarker for the diagnosis of rheumatoid arthritis [33]; circ_0000745, circ_0001531, and circ_0001640 have good diagnostic potential for breast cancer [34]; and hsa_circ_0006470 is highly valued as a diagnostic biomarker for GC [35]. Circ1662 was found to promote colorectal cancer cell invasion and migration and was integrated as a new prognostic marker and therapeutic target for colorectal cancer metastasis [36]. In contrast, circDLC1, which is down-regulated in hepatocellular carcinoma cells, was found to be a promising prognostic marker for patients with the disease [37]. Finally, circMAPK1 may be a new therapeutic target in the treatment of GCC [38].

In this study, we identified that the expression of circRNA-0000081 was significantly related to patients’ survival time and had obvious cancer-promoting effects, especially when it was overexpressed. It should be noted that long-term follow-up of patients in this study to determine the relationship with our circRNA-0000081 and the results of the study on the positive and negative phenotypic function of circRNA-0000081 are the basis for our continued experiments on this circRNA. In other words, the basis for our further research on circRNA is more reliable than some studies [39,40].

The current study found that the role of circRNA in cancer is not independent from other molecules. This is consistent with the findings of previous studies that investigate the functions of circRNA in cancer, such as circ3823, which was found to be up-regulated in colorectal cancer, and it was shown to promote colorectal cancer growth, metastasis, and angiogenesis through the circ3823/miR-30c-5p/TCF7 axis [7]. Similarly, circWAC was highly expressed in triple-negative breast cancer, affecting the chemosensitivity of cells through the circWAC/miR-142/WWP1 network [41]. In addition, circFAM73A [42], circVAPA [43], circRNA_100876 [44], circPDZD8 [45], and circDONSON [46] played regulatory roles in the progression of GC by acting as molecular sponges of miRNA. Contextually, in our study, we found that miRNA-423-5p was adsorbed by circRNA-0000081, thus playing a tumor suppressor role in GC cells, and its function on GC cells was partially restored by circRNA-0000081. We also found that miRNA-423-5p could partially restore the function of circRNA-0000081 on GC cells.

We also know that miRNAs act in cells primarily through their effects on downstream targets. SidaZhao et al. found that miR-423-5p inhibited the expression of SYT1 and PTTG1 at the mRNA and protein levels, thus promoting tumorigenesis in somatotroph adenomas [47]. Along the same line, JingjingLiu et al. found that miRNA-423-5p negatively regulated the expression of TFF1, thus influencing cell proliferation and invasion in GC [48]. Therefore, in this study, PDPK1 was predicted as a downstream target gene of miRNA-423-5p by bioinformatics software. Furthermore, we found that PDPK1 was negatively correlated with miRNA-423-5p at both the mRNA and protein levels but positively correlated with circRNA-0000081. The effect of miRNA-423-5p on PDPK1 could be partially recovered by circRNA-0000081. The partial effect of circRNA-0000081 on PDPK1 was also partially restored by miRNA-423-5p. There are several reports that show that PDPK1 is associated with prostate cancer [49], neuroinflammation [50], autophagosome biogenesis [51], and also GC [52]. However, no studies have reported the correlation between PDPK1 and circRNA. So, our study provides novel insights regarding the role PDPK1 plays in cancer pathogenesis and progression.

**Figure 6.**
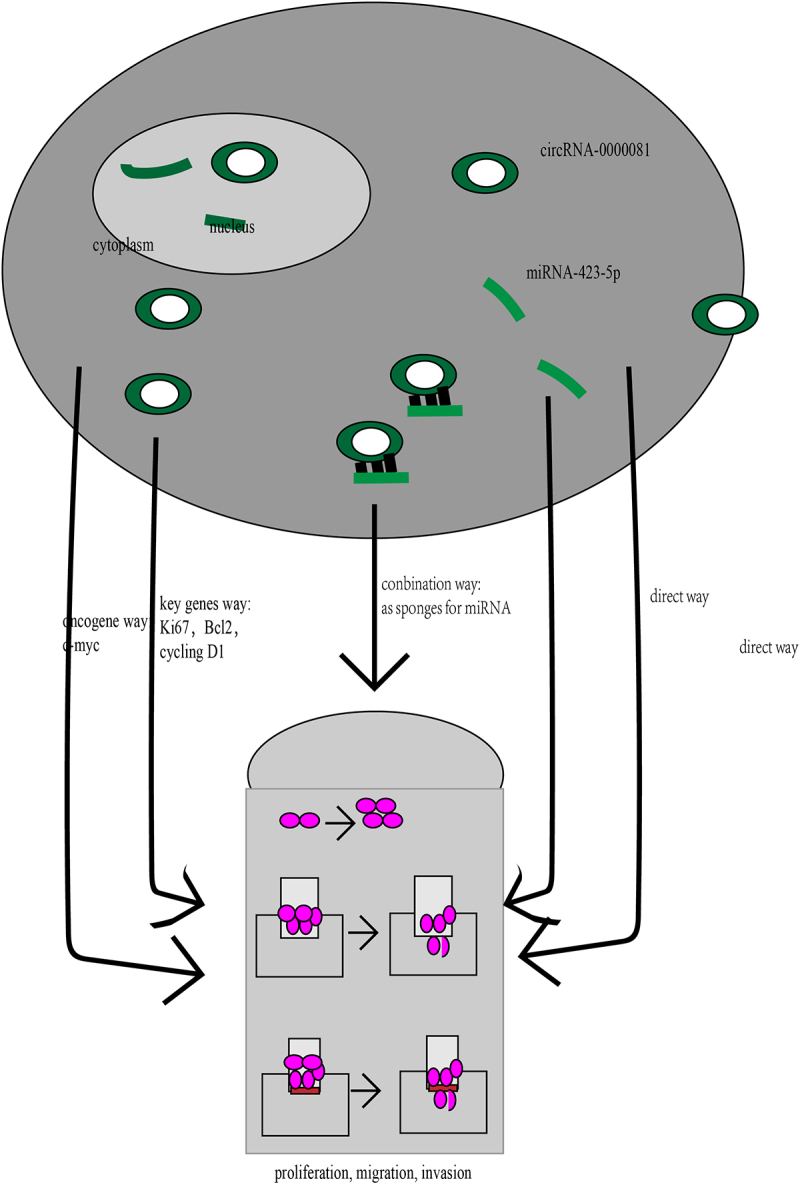
Several mechanisms by which circRNA-0000081 functions in gastric cancer cells.

In addition, circRNA-0000081 was found to affect the expression of Ki67, Bcl2, cyclin D1, and c-myc in vitro, which are key genes involved in proliferation [53], apoptosis [54], cell cycle [55], and oncogenes [56], respectively. This further supports the potential that circRNA-0000081 carries as a future therapeutic target (Figure 6a).

However, this study also has some limitations, such as the small sample size, which directly affects the reliability of the results. The relationship between circRNAs and miRNAs has been explored in detail, but the function of downstream genes and their relationship with circRNAs have not been thoroughly explored. Moreover, the investigation of the exact underlying mechanisms and interactions requires further evidence from animal experiments.

Now, with the increasing number of studies on the role of circRNA in diseases [9^,^^11^^,^^37^^,57–60^], research on the role circRNA in several diseases [^61–63^] and its potential role as a therapeutic target are attracting the interest of several research groups around the globe [64,67]. The utilization of circRNA in future drug discovery missions and their integration into future treatment regimens might be the future of this field.

## 5 Conclusion

In this study, we found that circRNA-0000081 expression was up-regulated in GC tissues. We have also explored the underlying interaction with miRNA-423-5p and regulation of the expression of PDPK1, Ki67, Bcl2, cyclin D1, and c-myc. All these findings could be the basis for designing new drugs or technologies that could target circRNA-0000081 and provide better outcomes in the treatment of GC.

## Supplementary Material

Supplemental Material

## Data Availability

All data are included in the article.
