# Supplementary figures and images for: Hsa_circ_0000081 promotes the function of gastric cancer through sponging hsa-miR-423-5p to influence 3-phosphoinositide-dependent kinase 1 expression
Source: Bioengineered. 2022 Mar 18;13(4):8277–90. doi: 10.1080/21655979.2022.2053796 (PMC9162021; doi:10.1080/21655979.2022.2053796)

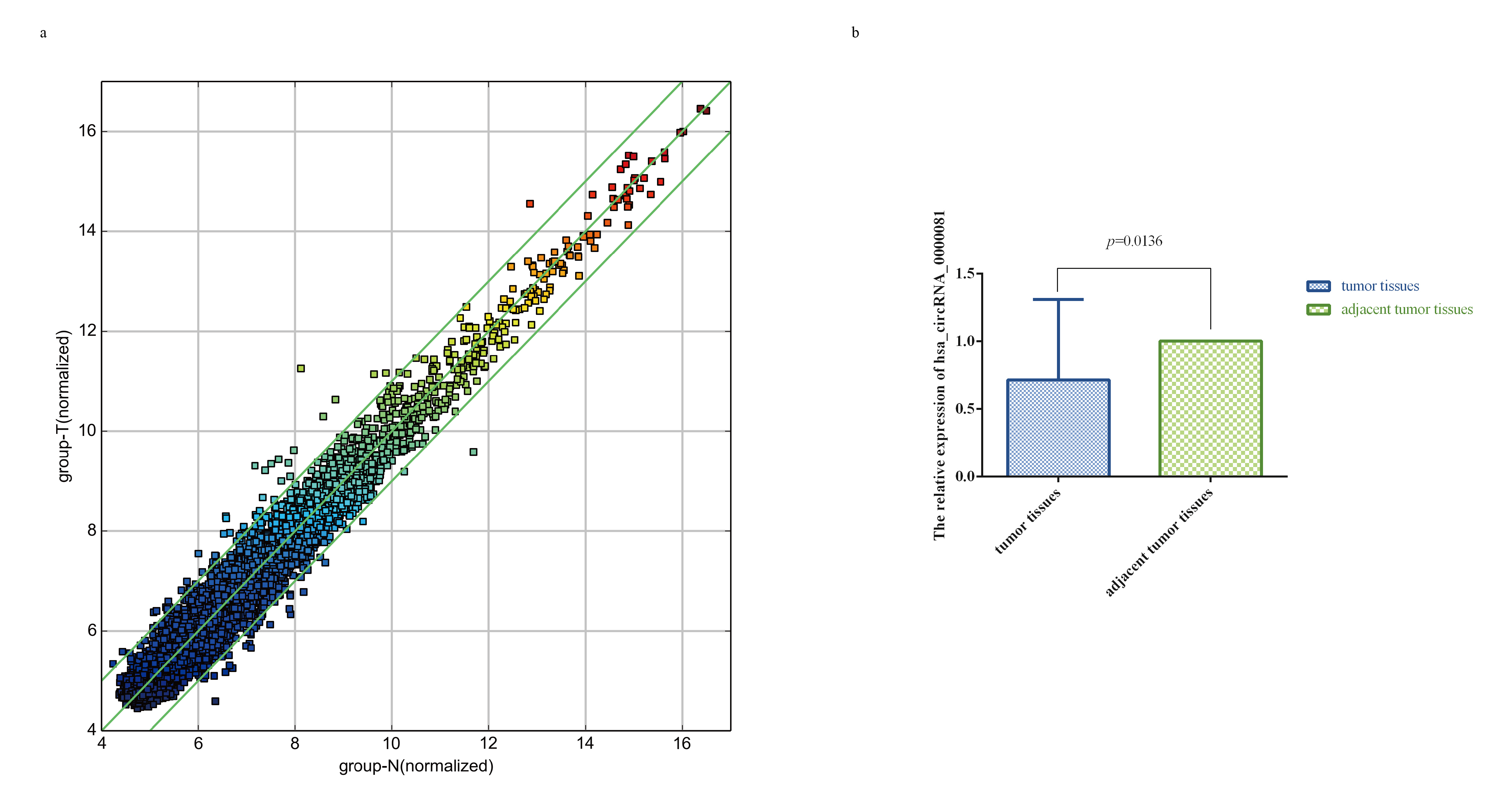

Supplement: Supplemental Material [file KBIE_A_2053796_SM7566.zip › Fig S1.jpg]

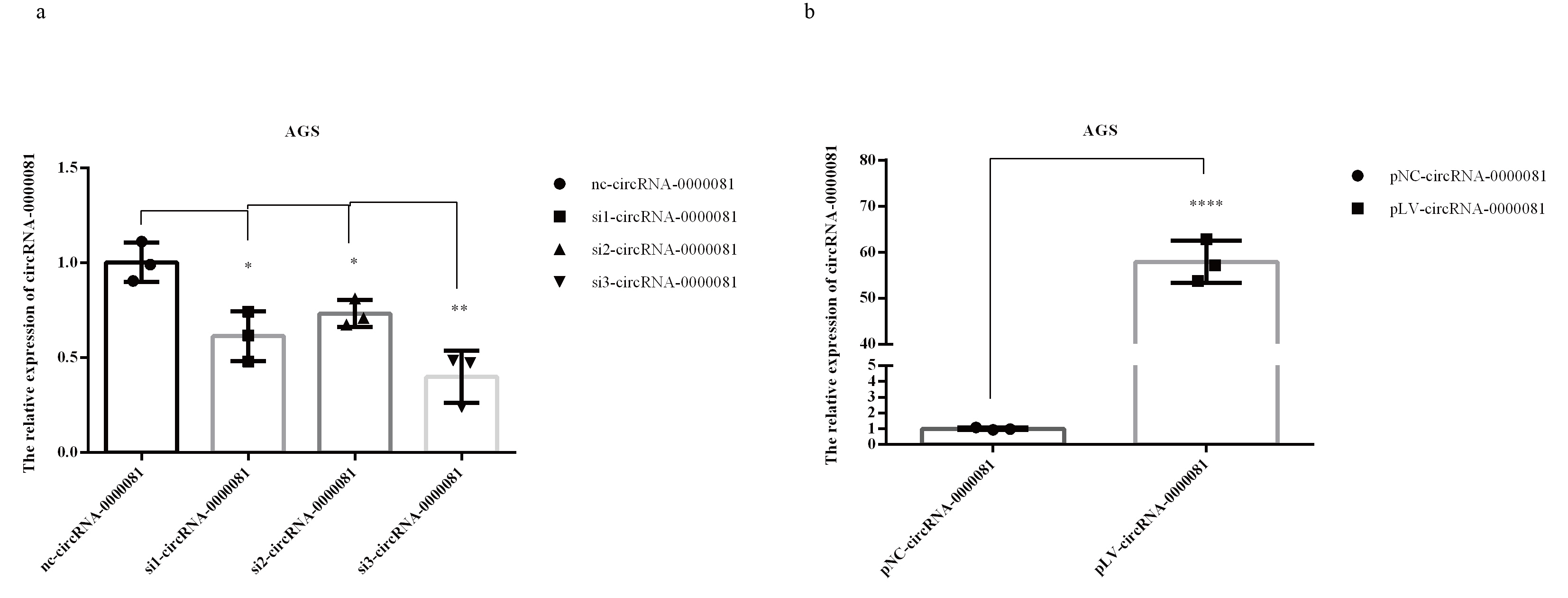

Supplement: Supplemental Material [file KBIE_A_2053796_SM7566.zip › Fig S2.jpg]

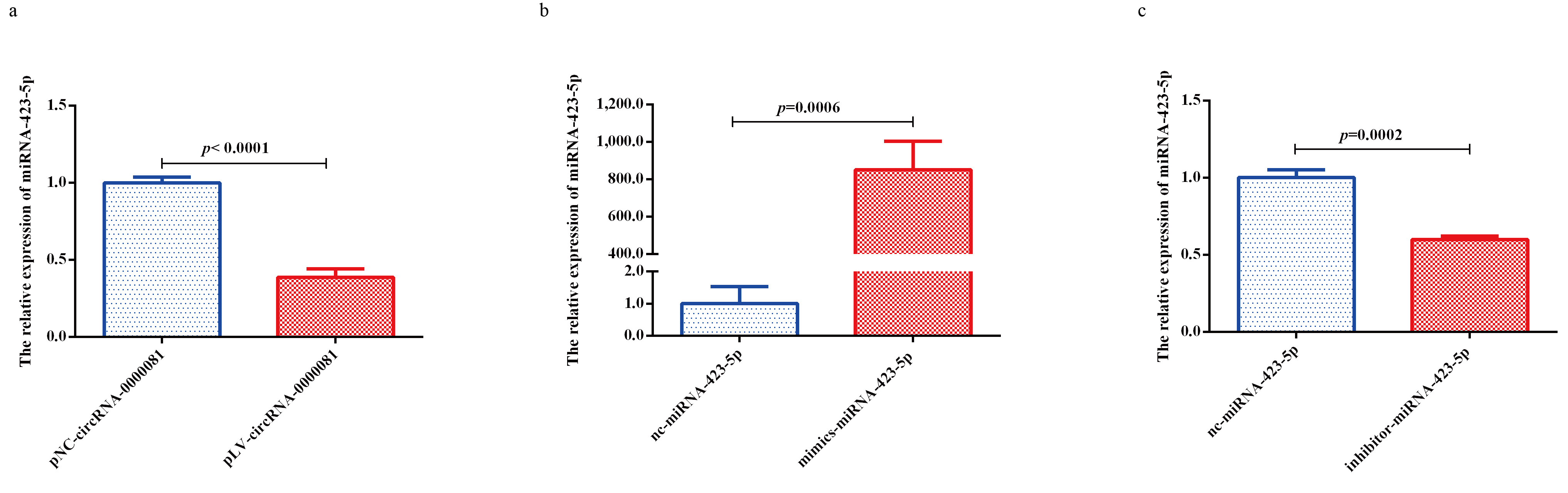

Supplement: Supplemental Material [file KBIE_A_2053796_SM7566.zip › Fig S3.jpg]
